# Identification and Analysis of Components in Yizhi Granule and Cynomolgus Monkey Plasma after Oral Administration by UPLC/ESI-Q-TOF MS and Their Protective Effects on PC12 Cells

**DOI:** 10.1155/2020/5165631

**Published:** 2020-04-09

**Authors:** Erwei Hao, Jianfeng Qin, Wei Wei, Jianhua Miao, Yan Xie, Xianglong Pan, Hangxuan Wu, Jinling Xie, Xiaosu Fan, Zhengcai Du, Xiaotao Hou, Jiagang Deng

**Affiliations:** ^1^Guangxi Key Laboratory of Efficacy Study on Chinese Materia Medica, Guangxi University of Chinese Medicine, Nanning, Guangxi 530200, China; ^2^Collaborative Innovation Center for Research on Functional Ingredients of Agricultural Residues, Guangxi University of Chinese Medicine, Nanning, Guangxi 530200, China; ^3^Postdoctoral Workstation, Guangxi Institue of Medicinal Plants, Nanning 530023, China; ^4^Experimental Center of College of Agriculture, Guangxi University, Nanning 530005, China

## Abstract

Yizhi Granule (YZG) is a health food containing six traditional Chinese medicines (TCMs). It improves memory barriers in rat experiments. Here, we describe the first fast and sensitive ultraperformance liquid chromatography/electrospray ionization quadrupole time-of-flight mass spectrometry (UPLC/ESI-Q-TOF MS) method for analyzing YZG in plasma. We used this technique for studies in cynomolgus monkey plasma. By comparing retention time, MS, and MS/MS data of reference compounds, 70 compounds were detected in YZG. Of these, 63 were identified including 60 saponins, 2 flavones, and 1 methyl ester. There were 33 saponins, 1 flavone, and 1 methyl ester in the plasma. Next, to study the therapeutic properties of YZG, the neuroprotective effect of some of the absorbed components was evaluated using PC12 cell damage caused by the A*β*_25–35_ model. The results showed that 9 compounds protect PC12 cells from A*β*_25–35_ with cell viability (%) of 111.00 ± 8.12 (G-Rb1), 102.20 ± 4.22 (G-Rb2), 100.34 ± 6.47 (G-Rd), 102.83 ± 2.10 (G-Re), 101.68 ± 7.64 (NG-Fa), 101.19 ± 7.83 (NG-R1), 102.53 ± 0.55 (NG-R2), 106.88 ± 4.95 (gypenoside A), and 103.95 ± 4.11 (gypenoside XLIX), respectively, versus the control group (87.51 ± 6.59). These results can reveal the real pharmacodynamic basis of YZG and provide a theoretical basis for subsequent studies. It can also provide some references for the research of Alzheimer's disease.

## 1. Introduction

Alzheimer's disease (AD) is a common chronic progressive neurodegenerative disease leading to memory impairment, hypophrenia, behavioral personality degeneration, disability, and premature death [1–3]. The prevalence of AD in China was 3.21% among people aged 65 years and older [4], and more than 7 million Chinese people live with AD today [5]. AD is not only a serious health problem for the elderly but also a severe social problem. It is of global concern. Therefore, developing new drugs to prevent and treat AD is critical. The amyloid hypothesis is the dominant model of AD pathogenesis and guides the development of potential treatments. All AD patients undergo progressive *β*-amyloid deposition followed by surrounding neuritic and glial cytopathology in brain regions serving memory and cognition [6].

Traditional Chinese medicine (TCM) plays a vital role in treating Alzheimer's disease in China. It is an essential source for new drug development. Many components of Chinese herbs such as ginsenoside Re (G-Re) [7], G-Rg3 [8], Baicalin, G-Rb1 [9], G-Rg1 [10], and G-Rf [11] were studied and shown to have an active function on treating AD. YZG is a health food composing 6 TCMs, including *Panax ginseng* C.A. Mey, *Panax notoginseng* (Burk) F. H. Chen, *Gynostemma pentaphyllum* (Thunb.) Makino, *Epimedium brevicornu* Maxim., *Alpinia oxyphylla* Miq., and *Morus alba* L. It was approved as a health food by the CFDA with 2 Chinese patents issued in December 2012.

We recently showed that YZG improved memory barriers in animal experiments, and YZG could protect the PC12 from the damage induced by protein A*β*_25-35_ [12–15]; however, the active ingredients remain unclear [16]. Although TCMs contain many components, only some are absorbed into the blood for biological activity. Therefore, components found in the plasma are likely to be the most active.

Cynomolgus monkeys are nonhuman primates and are similar to humans in genetics and pathophysiology; thus, they are a useful preclinical model [17, 18]. Therefore, we used this model to evaluate the TCMs.

**Figure 1:**
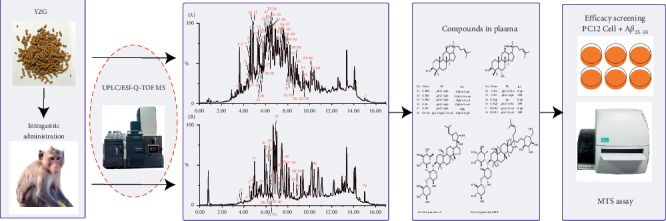
Experimental design and workflow in this study.

TCMs exert their effects through multiple components. Analysis of the chemical composition is a crucial step to understand the therapeutic properties of TCMs. Ultraperformance liquid chromatography-mass spectrometry (UPLC-MS) method has become a dominant tool to analyze the chemical components of TCMs because it offers high speed, wide measurable mass range, high ratio of the resolution, and the capacity for simultaneous qualitative analysis, which is also widely applied in the analysis of *in vivo* metabolites. For instance, UPLC-QTOF-MS technique was used to identify the absorbed constituents and their metabolic products in rat biosamples [19], and in their following study, an ESI/APCI multimode ionization source was used for LC-MS analysis in order to identify saponin glycosides and saponin aglycones in a single run [20]. In other studies, based on the LC-DAD-MS and UPLC–DAD–QTOF-MS methods, chemical composition, metabolism, and pharmacokinetic studies of herbal medicines were also conducted [21, 22]. Hence, we established a UPLC/ESI-Q-TOF MS method for the analysis of the chemical composition of YZG and the absorbed ingredients in the plasma of cynomolgus monkeys. Furthermore, the AD cell model induced by *β*-amyloid was used to test the protective effects of the absorbed components on nerve cells. The experimental design and workflow of this study were summarized in Figure 1.

## 2. Materials and Methods

### 2.1. Chemicals, Reagents, and Samples

UPLC-grade acetonitrile was purchased from Merck (Darmstadt, Germany). Formic acid was purchased from Sigma-Aldrich (Mo, USA). Leucine enkephalin was obtained from Waters Corporation (Milford, MA, USA). Distilled water was obtained from Watson's Food & Beverage (Guangzhou, China).

**Figure 2:**
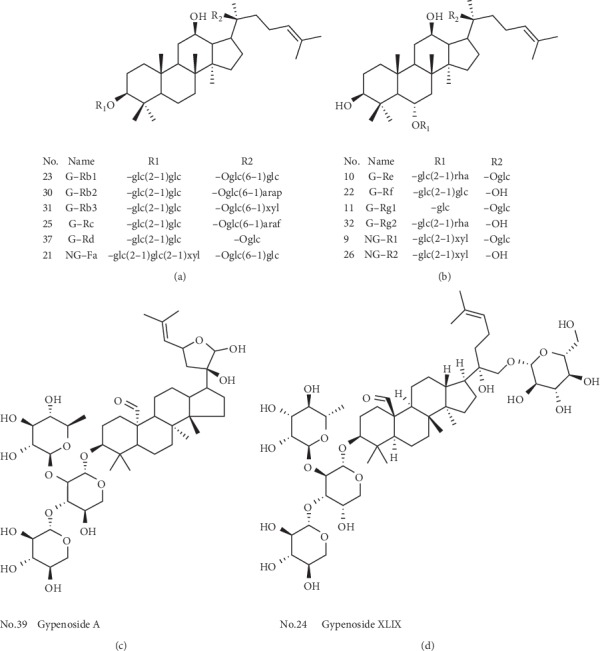
Chemical structures of the possible effective compounds of YZG.

The PC12 cells were purchased from Jiangsu KeyGEN BioTECH Corp., Ltd (Nanjing, China). Ginsenoside Rb1, ginsenoside Rb2, ginsenoside Rb3, ginsenoside Rc, ginsenoside Rd, ginsenoside Re, ginsenoside Rf, ginsenoside Rg1, notoginsenoside R1, 20(*S*)-notoginsenoside R2, notoginsenoside Fa, gypenosides A, and gypenosides XLIX were purchased from Chengdu Must Bio-Technology Co., Ltd. (Chengdu, China). The 20(*S*)-ginsenoside Rb2, galantamine HBr, and berberine were purchased from the National Institutes for Food and Drug Control (Beijing, China). The structure of these compounds is shown in Figure 2. Dulbecco's modified Eagle medium (DMEM) and phosphate buffer saline (PBS) were purchased from Jiangsu KeyGEN BioTECH Corp., Ltd. (Nanjing, China). Fetal bovine serum (FBS), trypsin, and dimethyl sulfoxide (DMSO) were purchased from Gibco (Thermo Fisher Scientific, Inc., Waltham, MA, USA). Amyloid *β*-protein fragment 25–35 was purchased from Sigma-Aldrich (St. Louis, MO, USA). YZG was produced by Guangxi Wanshoutang Pharmaceutical Co., Ltd., and formulated by water into suspension.

### 2.2. Animals and Treatments

Six healthy male cynomolgus monkeys (*Macaca fascicularis*; 7 years old, 7.0 ± 1.0 kg) were obtained from Guangxi cynomolgus medicine applied engineering technology research center (Guangxi province). All experiments were conducted in accordance with the Regulations of Experimental Animal Administration issued by the State Commission of Science and Technology of the People's Republic of China. Experimental animal protocols were approved by the Animal Ethics Committee of the Guangxi University of Chinese Medicine, and all procedures were following the relevant regulations and guidelines.

Each monkey was housed in a suspended stainless steel cage and was maintained under a standard 12 h light/12 h dark cycle with free access to water. Animal rooms were kept at 24–26°C and relative humidity of 50%–70%. A certified primate pellet diet was provided to the monkeys three times each day before the experiments. Fruits were supplemented regularly for nutrition as is standard practice.

### 2.3. Plasma Sample Preparation

Six cynomolgus monkeys were fasted, except water, for 12 hours. Each monkey was orally administered YZG at a dose of 79.6 g/kg body weight. 2 mL of blood was collected by venipuncture 2 h after dosing via intragastric gavage. The blood was then centrifuged for 10 min at 3000 rpm/min at 4°C. Methanol (3 mL) was put in the plasma and vortexed for 1 min and then was centrifuged for 10 min at 12000 rpm/min at 4°C. The supernatant was purified by solid-phase extraction. The purified liquid was dried under nitrogen gas at 45°C. The residues were dissolved in 100 *μ*L of 70% methanol and then centrifuged at 12000 rpm/min for 10 min at 4°C; the supernatant was used as the plasma sample.

### 2.4. Instrument and Conditions

#### 2.4.1. Chromatographic Analysis

The separation process was performed by the Waters ACQUITY UPLC I-Class system (Waters Corporation, Milford, MA, USA) with the controlled software of Masslynx V4.1. The mobile phase consisted of solvent A (HCOOH : H_2_O = 0.1 : 100, *v/v*) and solvent B (CH_3_CN); the gradient eluting procedure was as follows: 0-1 min, 10%B; 1–14 min, 10%–100%B; and 14–17 min, 100% B at a flow rate of 0.3 mL/min. The volume of the sample solution injected into the chromatographic system was 1 *μ*L, and all the separations were performed at 40°C.

#### 2.4.2. MS Conditions

The MS analysis was performed by the Xevo G2-XS time-of-flight mass spectrometer coupled with an ACQUITY UPLC I-Class system (Waters Corporation, Milford, MA, USA). The ESI-MS/MS experiment was operated in MS^E^ mode to obtain fragmentation in the negative mode. MS conditions were optimized as follows: the acquisition mass range was from 100 to 1500 Da with a 0.5 s scan time; capillary voltage was 2.0 kV; sampling cone voltage was 50.0 V; source temperature was 100°C; desolvation temperature was 350°C; cone gas flow was 50.0 L/Hr; desolvation gas flow was 700.0 l/Hr. The data were collected on a continuum, and the mass was corrected during acquisition using an external reference (LockSpray) consisting of 0.2 ng/mL solution of leucine enkephalin infused at a flow rate of 20 *μ*L/min via a LockSpray interface generating a reference ion at 554.2615 Da ([M-H]^−^). All data collected in centroid mode were acquired using MassLynx V4.1 software (Waters Corporation, Milford, MA, USA).

### 2.5. Principal Component Analysis (PCA) and Orthogonal Partial Least Squares Discriminant Analysis (OPLS-DA)

The original data peak detection of UPLC-TOF MS, principal component analysis (PCA), and orthogonal partial least squares discriminant analysis (OPLS-DA) were performed on all blood samples by using the MarkerLynx Application Manager in MassLynx V4.1 software. The quality window of peak detection was 0.05, the retention time window was 0.2, and the strength threshold was 50. The Pareto method was used as the data standardization method for PCA and OPLS-DA. Then, we selected the signals from the S-plot figure of OPLS-DA which meet the following conditions: *X*-axis >0.001 and *Y*-axis >0.8 as the marker.

### 2.6. Identification of the Absorbed Components

We compared the retention time and mass data of the markers with the data of medicinal herbs and selected the data that matches. At the same time, we searched the original ingredients in the medicinal herbs in the mass spectrometry data of the plasma sample. Then, we analyzed the secondary mass spectrometry information of these data and identified their chemical structures based on the information provided by the fragment ions.

### 2.7. Cell Culture and MTS Colorimetric Assay

PC12 cells were cultured in DMEM (Jiangsu KeyGEN BioTECH Corp., Ltd; Nanjing, China) supplemented with 10% FBS, at 37°C with 5% CO_2_. PC12 cells in the logarithmic growth phase were seeded in a 384-well plate (3 × 10^4^ cells per well) and incubated at 37°C for 24 h. Next, 100 *μ*L of the blank medium was added to the normal control group; 100 *μ*L of medium containing 30 *μ*M A*β*_25–35_ was added to the model control group; 100 *μ*L of medium containing 30 *μ*M A*β*_25–35_ and 250 *μ*g/mL YZG extracting solution was added to YZG group. The galantamine hydrobromide group received 100 *μ*L of medium containing 30 *μ*M A*β*_25-35_ and 30 *μ*g/mL galantamine hydrobromide. The berberine hydrochloride group received 100 *μ*L medium containing 30 *μ*M A*β*_25–35_ and 12.5 *μ*g/mL berberine hydrochloride. The other groups treated with A*β*_25–35_ and part of the absorbed components were separately given 100 *μ*L of medium containing 30 *μ*M A*β*_25–35_ and 25 *μ*g/mL component. All groups were incubated for 48 h. The volume fraction of DMSO in each group was not higher than 0.5%, and 3 duplicate wells were set in each group. Subsequently, 10 *μ*L MTS (1.90 mg/mL) was added to each well and incubated at 37°C for 24 h. The absorbance was measured at 480 nm using a spectrophotometer (Multimode Plate Reader EnVisionXcite; PerkinElme, Inc., Waltham, Massachusetts, USA). Cell viability was determined using the following equation: cell viability (%) = [OD 480 nm (drug)/OD 480 nm (control)] × 100%. OD indicates optical density.

### 2.8. Statistical Analysis

All data are presented as the mean ± standard error of the mean from at least three independent experiments. Data analysis was performed using GraphPad Prism 5.0 software (Graphpad Software, Inc., La Jolla, CA, USA). Statistical significance was also determined via a two-way analysis of variance.

## 3. Results

### 3.1. Optimization of UPLC/ESI-Q-TOF MS Conditions

To resolve the YZG components, the column type, temperature, and mobile phase were optimized. An ACQUITY UPLC BEH C18 column (2.1 mm × 100 mm, 1.7 *μ*m, Waters Corporation, USA) was selected for the experiment. It offered a good resolution. To minimize peak width and maximize signal intensity, organic solvents including acetonitrile and methanol, several aqueous buffers, flow rate, and column temperature were investigated. Finally, optimal separation conditions were obtained. The MS conditions were optimized to maximize the response: capillary voltage, capillary temperature, collision energy, and gas flow. In negative ionization mode, all YZG analytes (extract and plasma) showed high sensitivity.

### 3.2. UPLC/ESI-Q-TOF MS Analysis of the Ingredients in YZG Samples and Cynomolgus Monkey Plasma

**Figure 3:**
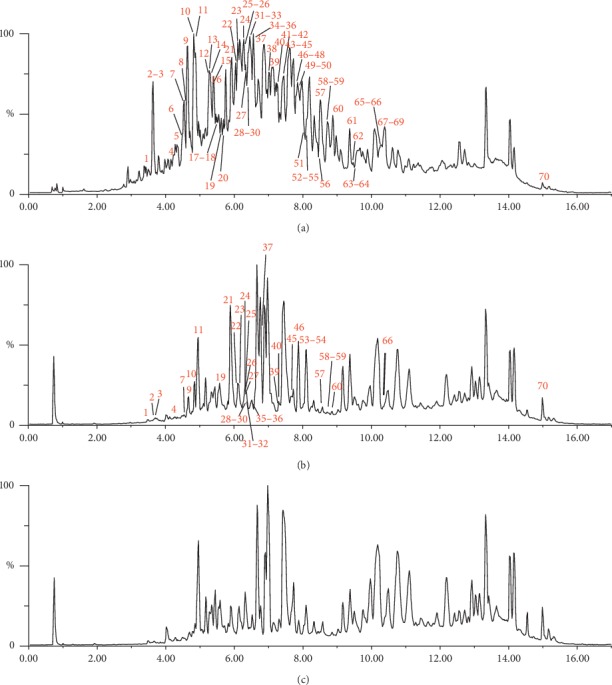
TIC chromatograms of YZG (a), the sample in cynomolgus monkeys' plasma after oral administration of YZG (b), and blank plasma in negative mode (c).

The total ion current for the YZG samples is shown in Figure 3. Seventy peaks were detected in YZG using the UPLC/ESI-Q-TOF MS technique; 63 compounds were structurally identified via comparison of retention time, MS, and MS/MS data of the reference compounds and those reported in the literature. The identified compounds are shown in Table 1.

To identify the plasma components, the MS data of the drug plasma and blank plasma were compared using the MarkerLynx module in the MassLynx (V4.1) software.

**Figure 4:**
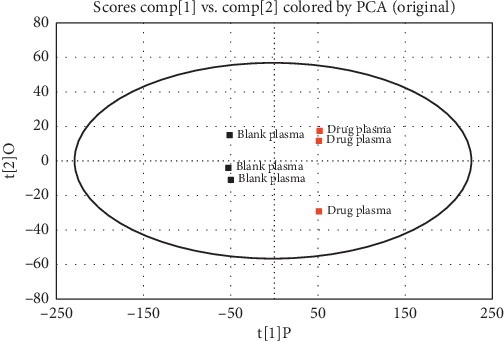
Score plot from principal component analysis (PCA) of drug plasma and the blank plasma.

The PCA scores of the UPLC/ESI-Q-TOF MS data of the drug plasma and the blank plasma were shown in Figure 4. It can be seen from the score chart that the scores of the drug plasma and the blank plasma differed significantly from each other in terms of the first principal component scores, showing significant differences in the data of the two groups and suggesting significant differences in the chemical components of the two groups.

**Figure 5:**
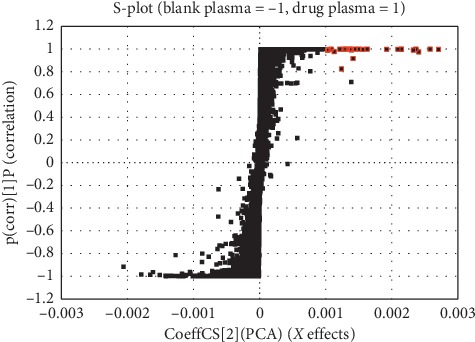
S-plot from orthogonal partial least squares discriminant analysis (OPLS-DA) of drug plasma and the blank plasma.

The S diagram (S-plot) of OPLS-DA for the UPLC/ESI-Q-TOF MS data of drug plasma and blank plasma is shown in Figure 5. Each point in the S-plot represents a data signal, the horizontal coordinate represents the contribution degree, the vertical coordinate represents the credibility, and the positive direction of the coordinate axis represents the signal of the drug group which is stronger than that of the blank group. The signal selected in the S-plot conforms to the following criteria: *X* > 0.001 and *Y* > 0.8.

**Figure 6:**
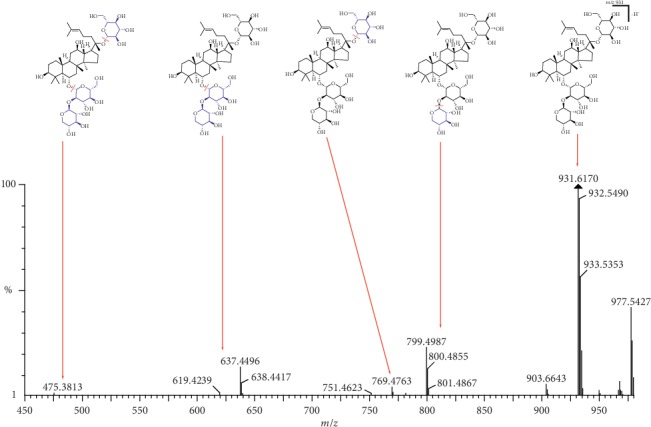
LC-MS spectrum and fragmentation pathway of notoginsenoside R1.

**Figure 7:**
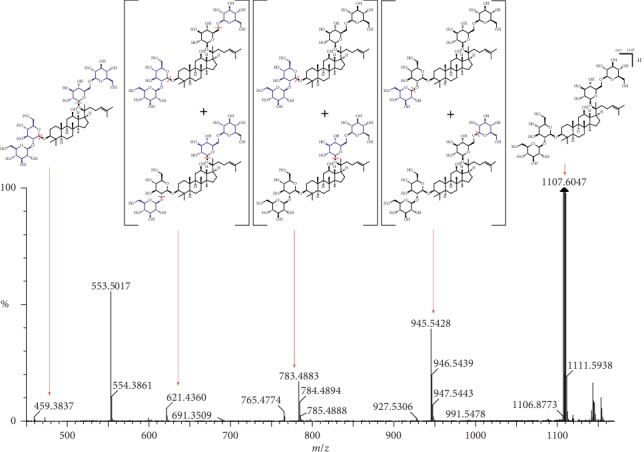
LC-MS spectrum and fragmentation pathway of ginsenoside Rb1.

**Figure 8:**
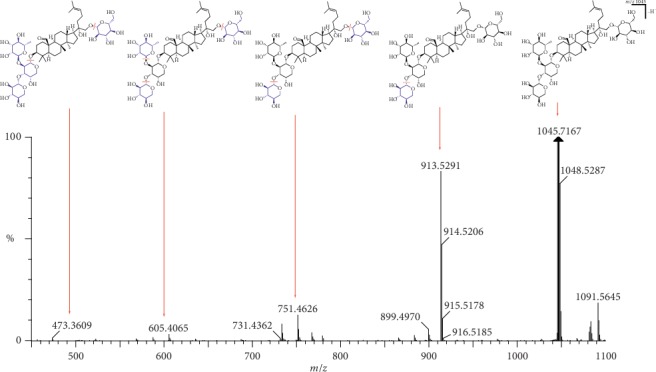
LC-MS spectrum and fragmentation pathway of gypenoside XLIX.

**Figure 9:**
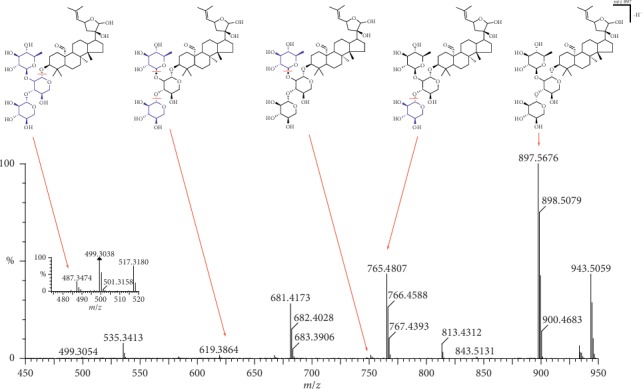
LC-MS spectrum and fragmentation pathway of gypenoside A.

36 compounds were found and structurally identified (Table 1). The MS/MS spectra and fragmentation pathways of some absorbed components in plasma are shown in Figures 6–9.

Peak 9 (Rt 4.61 min) produced [M-H]^−^ ions at *m*/*z* 931.5317 and [M + HCOO]^−^ ions at *m*/*z* 977.5385 indicating that the molecular formula was C47H80O18. Its MS/MS data showed characteristic fragments formed at *m*/*z* 799.4850 and 637.4321 as shown in Figure 6. This suggests the loss of one xylosyl group followed by one glycosyl group. The mass fragmentation behavior of this compound suggested that it was notoginsenoside R1, which was confirmed by comparison to the literature [26].

Peak 23 (RT 6.11 min) produced [M-H]^−^ ions at *m/z* 1107.6023 indicating that the molecular formula was C_54_H_92_O_23_. The MS/MS data *m/z* 945.5431 (Figure 7) suggests the loss of one glycosidic group and *m/z* 783.4896 suggests one more glycosidic group loss. In addition, the MS/MS data indicated the loss of another two glycosidic groups, which are shown at *m/z* 621.4235 and *m/z* 459.3650, respectively. The mass fragmentation behavior of this compound suggested that it could be identified as ginsenoside Rb1 as confirmed by comparison to the reported data.

Peak 24 (Rt 6.21 min) produced a significant [M-H]^−^ ion at *m*/*z* 1045.7167 and [M + HCOO]^−^ ions at *m*/*z* 1091.5645. The molecular formula (C_52_H_86_O_21_) could be deduced via elemental composition. The characteristic fragments formed were at *m*/*z* 913.5291, 751.4626, 605.4065, and 473.3609, which were consistent with the standard compound. Thus, peak 24 was identified as gypenoside XLIX, and its fragmentation pathway is shown in Figure 8.

Peak 39 (RT 7.21 min) produced the [M-H]^−^ ion at *m/z* 897.5676 and [M + HCOO]^−^ ion at *m/z* 943.5059 indicating that the molecular formula was C_46_H_74_O_17._ The characteristic fragments formed were at *m/z* 765.4807, 751.4272, 681.4173, 619.3864, and 487.3429. The fragments at *m/z* 765.4807 and 751.4272 indicated the loss of one xylosyl group and one rhamnosyl group, respectively; the characteristic fragments at *m/z* 681.4173 indicated the loss of both. The fragmentation pathway of peak 39 is shown in Figure 9.

### 3.3. Pharmacological Action of Absorbed Components

The pathological features of Alzheimer's disease mainly include senile plaque (SP) formed by the deposition of *β*-amyloid (A*β*) outside the neurons as well as neurofibrillary tangles formed by hyperphosphorylation of tau protein in neurons, neufibrillary tangles (NFTs), and neuronal loss [17]. Excessive deposition of A*β* can induce oxidative stress; result in excessive accumulation of free radicals; lead to peroxidative damage of biomacromolecules lipids, proteins, DNA, and RNA [37]; and cause neuronal apoptosis [38]. Therefore, A*β*-induced oxidative stress plays a vital role in the pathogenesis of AD [38, 39]. Neuroinflammation is also one of the pathological features of AD [40] and is an essential mediator of A*β*-induced neuronal death and another essential factor in the induction of AD pathology in addition to oxidative stress [41]. There is increasing evidence that A*β*-induced inflammatory responses are an essential component of A*β* neurotoxicity [42]. PC12 cells are clonal cell lines of rat adrenal chromaffin cells. They are neurogenic and have typical neuroendocrine cell characteristics. They are widely used in the study of neuronal differentiation, ion channels, receptors, and transmitter secretion. They are also one of the most common cell lines for studying neurotoxicity and are useful *in vitro* cell models [43]. Therefore, the active fragment A*β*_23–35_ was used in this experiment to induce PC12 cells to establish a neuronal injury model.

**Figure 10:**
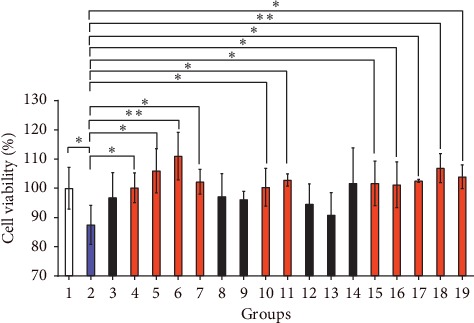
Screening of the components with a protective effect on PC12 treated with A*β*25-35. Samples are as follows: 1, blank control group; 2, model control group; 3, YZG group; 4, galantamine hydrobromide group; 5, berberine hydrochloride group; 6, ginsenoside Rb1 group; 7, ginsenoside Rb2 group; 8, ginsenoside Rb3 group; 9, ginsenoside Rc group; 10, ginsenoside Rd group; 11, ginsenoside Re group; 12, ginsenoside Rf group; 13, ginsenoside Rg1 group; 14, ginsenoside Rg2 group; 15, notoginsenoside Fa group; 16, notoginsenoside R1 group; 17, notoginsenoside R2 group; 18, gypenoside A group; and 19, gypenoside XLIX group.

After treating with A*β*_25–35,_ the PC12 cells in the model control group were obviously damaged—the cell viability of the model control group obviously decreased (compared with the blank control group, *p* < 0.05). Compared with the model control group, the positive control drugs galantamine and berberine could obviously protect PC12 cells from the damage of A*β*_25–35_(*p* < 0.05). Ginsenoside Rb1, ginsenoside Rb2, ginsenoside Rd, ginsenoside Re, notoginsenoside Fa, notoginsenoside R1, notoginsenoside R2, gypenoside A, and gypenoside XLIX have apparent protective effects on PC12 cells from the damage of A*β*_25–35_(*p* < 0.05) as shown in Figure 10. Table 1 shows that 36 compounds of YZG were absorbed in cynomolgus monkey plasma; however, only 14 of these compounds were used for pharmacological experiments into the neuroprotection effects. The other absorbed compounds in cynomolgus monkey plasma will be separated or purchased for the pharmacodynamic screening of neuroprotection effects. The active components in Figure 10 will be further verified in zebrafish or mouse models.

## 4. Discussion

YZG is a health food containing six TCMs. We recently showed that YZG improved memory barriers in animal experiments and YZG could protect the PC12 from the damage induced by protein A*β*_25–35_ [12–15]. The related effects of these six herbs in YZG have also been reported. Ginsenosides can reduce the formation of amyloid *β*-protein (A*β*) through inhibiting *β*- and *γ*-secretase activity or by activating the nonamyloidogenic pathway, inhibit acetylcholinesterase activity and induced neurotoxicity, and reduce the generation of reactive oxygen species and neuroinflammatory response by A*β* [44, 45]. *Panax notoginseng* can regulate the expression of AD-related circRNAs to achieve the therapeutic effect on AD [46]. Gypenosides can significantly improve learning ability and memory in rats with LPS-induced brain dysfunction [47]. Icariin prevents amyloid beta-induced apoptosis via the PI3K/Akt pathway in PC-12 cells [48] and improves synaptic plasticity through the BDNF/TrkB/Akt pathway [49]. *Alpinia Oxyphylla* can improve spatial memory performance and downregulated expressions of *β*-secretase and accumulation of A*β*_1–42_ in brain tissues [50]. The root bark of *Morus alba*, as well as its isolated compounds, has shown the potent AChE-, BChE-, and BACE1-inhibitory activities [51]. However, the active ingredients of YZG remain unclear.

Although traditional Chinese medicine contains complex chemical components, only the compounds that can be absorbed into the blood can produce effects. Traditional Chinese medicine is mostly administered orally, and its active substances must be transported to the action target through the blood so as to have an effect. Therefore, the components contained in the serum after administration are the direct-acting substances of traditional Chinese medicine *in vivo*. Cynomolgus monkeys are nonhuman primates and are similar to humans in genetics and pathophysiology; thus, they are a useful preclinical model [17, 18]. Therefore, we used this model to evaluate YZG. And the serum pharmacochemistry method was used to analyze and identify the components contained in the serum after oral administration of YZG in the cynomolgus monkeys.

In this study, a total of 63 compounds were determined from YZG using the UPLC/ESI-Q-TOF MS method; most of them were saponins come from *Panax ginseng* and *Panax notoginseng*, indicating that the main components of YZG that played a medicinal role were saponins. Then, we analyzed and identified the prototype components in the serum of cynomolgus monkey after the oral administration of YZG and found 35 prototype components. And we analyzed the cleavage rules of some representative components. This indicated that these compounds are the possible active components of YZG. In order to study whether these compounds really work, we used in vitro experiments to verify and purchased 14 commercially available compounds to do cell experiments; the results show that nine of them have significant pharmacological activities, which proves to a certain extent that the components absorbed into the blood are indeed the active components of YZG, which provides a basis for our later experiments. However, because other absorbed components are challenging to obtain, we did not verify all the absorbed components in this study, which is the deficiency of this experiment, and the follow-up study will find a way to solve this problem.

## 5. Conclusions

A rapid, sensitive, and convenient UPLC/ESI-Q-TOF MS method was established for the simultaneous qualitative analysis of the chemical compositions of YZG and the absorbed components in the plasma cynomolgus monkey. Seventy compounds were detected in YZG, and 63 compounds of these were identified including 60 saponins, 2 flavones, and 1 methyl ester. *In vivo* studies showed 33 saponins, 1 flavone, and 1 methyl ester in the plasma of cynomolgus monkeys. The PC12 cell damage model used A*β*25-35 to evaluate the neuroprotective effects of the absorbed components. The results showed that 9 compounds have protective effects: ginsenoside Rb1, ginsenoside Rb2, ginsenoside Rd, ginsenoside Re, notoginsenoside Fa, notoginsenoside R1, notoginsenoside R2, gypenoside A, and gypenoside XLIX. Most of these active components are saponins.

## Figures and Tables

**Table 1:** Constituents identified from YZG by UPLC-ESI-Q-TOF-MS.

No.	Rt	Formula	[M + HCOO]^−^ (*m*/*z*)	[M-H]^−^ (*m*/*z*)	Fragment ions (*m*/*z*)	CMP	Compound name	Reference
1	3.42	C_42_H_72_O_15_	861.4843	815.4784	653.4254, 491.2235, 463.0880	√	Notoginsenoside M	[23]
2	3.62	C_27_H_30_O_16_		609.1816	300.0555	√	Rutin	[24]
3	3.72	C_8_H_8_O_5_		183.0308	124.0211	√	Methyl gallate	[25]
4	4.22	C_48_H_82_O_19_	1007.5439	961.5380	997.5126, 799.4825, 637.4305, 475.1966	√	Notoginsenoside R3/R6/20-O-Glucoginsenoside Rf	[23]
5	4.28	C_40_H_70_O_8_	723.5082	677.5007	740.4865, 475.1963, 417.1550, 284.0329	^*∗*^	Unknown	
6	4.47	C_43_H_83_O_12_	836.5914	790.5905	853.5441	^*∗*^	Unknown	
7	4.55	C_48_H_82_O_19_	1007.5439	961.5406	997.5145, 799.4846, 637.4318, 475.3790	√	Notoginsenoside R3/R6/20-O-Glucoginsenoside Rf	[23]
8	4.57	C_32_H_38_O_15_	707.2444	661.2957	1323.5775, 724.2191, 499.1783	^*∗*^	Baohuoside A	∆
9	4.61	C_47_H_80_O_18_	977.5385	931.5317	799.4850, 637.4321, 475.3785	√	Notoginsenoside R1	[26]
10	4.81	C_48_H_82_O_18_	991.5449	945.5477	799.4883, 637.4339, 619.4214, 475.3792	√	Ginsenoside Re	[27]
11	4.85	C_42_H_72_O_14_	845.4936	799.4833	637.4339, 475.3792	√	Ginsenoside Rg1	[27]
12	5.24	C_39_H_50_O_20_	883.2853	837.2800	675.2280, 529.2005, 513.1791, 367.1183	^*∗*^	Epimedin A1	∆
13	5.29	C_15_H_10_O_7_		301.0683	273.0429, 245.0488, 229.0532, 179.0065, 151.0179, 121.0346	^*∗*^	Quercetin	∆
14	5.31	C_38_H_48_O_19_		807.2960	645.2173, 513.1818, 366.1090	^*∗*^	Epimedin B	∆
15	5.38	C_38_H_50_O_19_	867.2896	821.2642	659.2647, 513.1761, 366.1201, 351.0912, 323.0849, 151.0094, 106.6611	^*∗*^	Epimedin C	∆
16	5.40	C_38_H_50_O_19_	867.3390	821.2833	659.2668, 366.1226	^*∗*^	Epimedin A	∆
17	5.53	C_27_H_30_O_10_		513.1833	367.1188, 351.0876, 323.0917, 151.0109, 145.0312	^*∗*^	Isomer^(47)^	∆
18	5.53	C_33_H_40_O_15_	721.2323	675.2302	529.1712, 513.1846, 367.1266	^*∗*^	Icariin	∆
19	5.64	C_48_H_82_O_19_	1007.5430	961.5372	799.4822, 637.3738	√	Notoginsenoside N/R6	[28]
20	5.67	C_54_H_92_O_23_	1153.6027	1107.5963	945.5418, 799.4827, 637.4309, 475.1394,	^*∗*^	Ginsenoside Re8	[29]
21	5.90	C_59_H_100_O_27_	1285.6840	1239.6516	1107.5920, 945.5372, 783.4835, 621.4189, 459.3343	√	Notoginsenoside Fa	∆
22	6.09	C_42_H_72_O_14_	845.4875	799.4935	637.4294, 619.5074, 475.3772	√	Ginsenoside Rf	[28]
23	6.11	C_54_H_92_O_23_	1153.6823	1107.6227	945.5391, 783.4855, 621.4235, 459.3650	√	Ginsenoside Rb1	[30]
24	6.21	C_52_H_86_O_21_	1091.5645	1045.7167	913.5291, 751.4626, 605.4065, 473.3609,	√	Gypenoside XLIX	∆
25	6.26	C_53_H_90_O_22_	1123.5693	1077.6210	945.5375, 783.4875	√	Ginsenoside Rc	[30]
26	6.28	C_41_H_70_O_13_	815.4929	769.4709	637.4373, 619.4420, 475.3855	√	20(S)-Notoginsenoside R2	∆
27	6.33	C_41_H_70_O_13_	815.4812	769.4928	637.4339, 475.3815	√	Notoginsenoside R2/F3/F5	[28]
28	6.40	C_63_H_106_O_30_		1341.5354	1209.6248, 1077.5825, 945.5411, 783.4882, 621.4359, 459.3843	√	Notoginsenoside Q	[28]
29	6.41	C_48_H_76_O_19_		955.4913	793.4395, 569.3852	√	Ginsenoside rRo	[31]
30	6.42	C_53_H_90_O_22_	1123.5911	1077.5951	945.5395, 783.4940, 621.4150, 459.3434	√	Ginsenoside Rb2	[28]
31	6.44	C_53_H_90_O_22_	1123.5911	1077.5911	945.5429, 783.4907, 459.3839	√	Ginsenoside Rb3	[28]
32	6.46	C_42_H_72_O_13_	829.5134	783.5062	637.4319, 619.4190, 475.3806	√	20(S)-Ginsenoside Rg2	[30]
33	6.50	C_41_H_70_O_13_	815.4929	769.4709	637.4373, 619.4420, 475.3855	^*∗*^	20(R)-Notoginsenoside R2	∆
34	6.56	C_36_H_62_O_9_	683.4399	637.4317	475.3788	^*∗*^	20(S)-Ginsenoside Rh1/20(R)-Ginsenoside Rh1	[28]
35	6.56	C_42_H_72_O_13_	829.4958	783.4899	637.4317, 475.3788	√	20(R)-Ginsenoside Rg2	[30]
36	6.59	C_56_H_94_O_24_	1195.6116	1149.6077	1107.5959, 945.5424, 783.4896, 637.4297, 475.3786	√	Yesanchinoside F	[32]
37	6.85	C_48_H_82_O_18_	991.5511	945.5786	783.4921, 621.4363, 459.3667	√	Ginsenoside Rd	[28]
38	6.98	C_51_H_100_O_30_	1237.6125	1191.6177	1029.5610, 915.5285, 637.4301, 475.3689	^*∗*^	Unknown	
39	7.21	C_52_H_86_O_21_	943.5059	897.5676	765.4807, 751.4272, 681.4173, 619.3864, 487.3429	√	Gypenoside A	∆
40	7.26	C_55_H_92_O_23_	1165.6019	1119.5986	783.4898, 621.4372, 459.3844	√	Ginsenoside Rs2	[28]
41	7.44	C_27_H_30_O_11_	575.3054	529.1713	1105.6187, 1059.6130, 367.1176	^*∗*^	Icariside I	
42	7.49	C_47_H_80_O_17_	961.5392	915.5334	783.4893, 621.4371	^*∗*^	Notoginsenoside Fe/gynosaponin I/vinaginsenoside R16/R17	[28]
43	7.61	C_60_H_116_O_36_	1457.7361	1411.7128	1265.6560, 1133.6172, 987.5623, 841.4951, 475.3750	^*∗*^	Unknown	
44	7.62	C_48_H_82_O_18_	991.5460	945.5498	799.4841, 637.4338, 619.4188, 475.3750	^*∗*^	Unknown	
45	7.63	C_51_H_86_O_21_	1033.6028	987.5575	945.5498, 927.5349, 841.4951	√	Pseudoginsenoside Rc1	[28]
46	7.81	C_55_H_92_O_23_	1165.6125	1119.6310	1077.5851, 1059.5736, 945.5401, 927.5275, 765.4456, 621.3965, 459.2525	√	Ginsenoside Rs1	[28]
47	7.82	C_27_H_30_O_10_		513.1867	366.1149, 351.0916, 323.0886, 151.0092, 132.0236	^*∗*^	Baohuoside I	∆
48	7.82	C_50_H_98_O_29_	1207.6355	1161.6063	1175.6207, 1161.6063	^*∗*^	Unknown	
49	7.85	C_41_H_70_O_14_	831.4783	785.4698	653.4258	^*∗*^	Notoginsenoside Rw2	[33]
50	7.89	C_54_H_86_O_24_	1163.6464	1117.6188	1057.5961, 971.5587, 929.5478	^*∗*^	Hemsloside G2/ginsenoside ROA	[34]
51	8.05	C_42_H_70_O_12_	811.4849	765.4782	619.4163, 457.2605	^*∗*^	Ginsenoside Rg6	∆
52	8.14	C_52_H_104_O_30_	1253.6549	1207.6088	1207.6088, 1075.5676, 943.5283	^*∗*^	Unknown	
53	8.16	C_42_H_72_O_13_	829.4985	783.4911	637.4318, 475.3790	√	Ginsenoside Fc	[28]
54	8.17	C_51_H_86_O_21_	1033.5596	987.5541	945.5425 927.5313, 783.4911, 765.4796, 621.4370, 459.3835	√	Quinquenoside III	[28]
55	8.18	C_42_H_72_O_14_	845.4895	799.4831	637.4308, 619.4199, 475.3775	^*∗*^	Isomer^(22)^	[28]
56	8.42	C_48_H_82_O_16_	959.5573	913.5543	767.4940, 621.4166, 475.3465, 146.9997	^*∗*^	Gynosaponin II	[35]
57	8.50	C_56_H_94_O_24_		1149.6082	1107.7029, 945.5416, 927.5334, 783.4859, 765.3539, 621.4349, 459.3838	√	Quinquenoside R1	[28]
58	8.72	C_42_H_72_O_13_	829.4963	783.4920	767.4591, 621.4376, 459.3846	√	20(S)-Ginsenoside Rg3	[23]
59	8.77	C_42_H_72_O_13_	829.4974	783.4915	765.4796, 621.4368, 459.3842	√	Isomer^(58)^	[23]
60	8.86	C_42_H_72_O_13_	829.5020	783.4990	621.4348, 459.3762, 161.0500	√	20(R)-Ginsenoside Rg3	[23]
61	9.39	C_36_H_62_O_9_	683.4375	637.4319	475.3790	^*∗*^	20(S)-Ginsenoside Rh1/20(R)-Ginsenoside Rh1	[28]
62	9.54	C_47_H_80_O_18_	977.4802	931.5010	799.4818, 769.4988, 751.4977, 637.3975	^*∗*^	Isomer^(9)^	[28]
63	9.59	C_49_H_80_O_18_	1001.5699	955.5613	913.5525, 767.4947	^*∗*^	Gylongiposide I	[36]
64	9.60	C_47_H_80_O_17_	961.5573	915.5556	783.4860, 621.4335, 459.3809	^*∗*^	Notoginsenoside Fe/gynosaponin I/vinaginsenoside R16/R17	[28]
65	10.22	C_42_H_70_O_12_	811.4861	765.4803	619.4220, 603.4269, 457.2275	^*∗*^	Ginsenoside Rg6	[28]
66	10.26	C_42_H_72_O_13_	829.4875	783.4943	621.4368, 459.3837	√	Ginsenoside F2	[28]
67	10.36	C_42_H_70_O_12_	811.4857	765.4805	619.4229, 603.4269, 457.2340	^*∗*^	Ginsenoside F4	[28]
68	10.38	C_42_H_70_O_12_	811.4849	765.4792	603.3373	^*∗*^	Ginsenoside Rg5	[28]
69	10.40	C_42_H_70_O_12_	811.4850	765.4794	603.3384, 483.2727	^*∗*^	Ginsenoside Rk1	[28]
70	15.05	C_47_H_80_O_17_	961.6223	915.5983	978.5970, 783.5145, 621.3875, 459.3448	√	Notoginsenoside Fe/gynosaponin I/vinaginsenoside R16/R17	[28]

Abbreviations: Rt, retention time; CMP, cynomolgus monkeys plasma; Ref., references. √: compound detected; ^*∗*^: compound not detected. isomer^(N^°^.)^: numbers in parentheses represent the peak corresponding to isomers; ∆: the data are consistent with the standard substance.

## Data Availability

The data used to support the findings of this study are included within the article.

## Abbreviations

YZG: Yizhi Granule
TCM: Traditional Chinese medicine
UPLC/ESI-Q-TOF MS: Ultraperformance liquid chromatography/electrospray ionization quadrupole time-of-flight mass spectrometry
AD: Alzheimer's disease
PCA: Principal component analysis
OPLS-DA: Orthogonal partial least square discrimination analysis
DMSO: Dimethyl sulfoxide
SP: Senile plaque
A*β*: *β*-Amyloid
NFTs: Neurofibrillary tangles.
